# A rapid in vitro method to flip back the double-floxed inverted open reading frame in a plasmid

**DOI:** 10.1186/s12896-018-0462-x

**Published:** 2018-08-31

**Authors:** Jian Xu, Yongling Zhu

**Affiliations:** Department of Ophthalmology, Northwestern University Feinberg School of Medicine, 303 E Chicago Ave, Tarry 5-723, Chicago, IL 60611 USA

**Keywords:** Cre, LoxP, Lox2272, FLEX, DIO

## Abstract

**Background:**

The LoxP site based genetic switch, the FLEX, also known as DIO (Double-Floxed Inverted Open reading frame), was invented to turn on gene expression via Cre-mediated recombination. Since its first publication, numerous FLEX switch plasmids have been generated. These plasmids are designed to only work in combination with Cre. However on many occasions it is necessary to covert these FLEX plasmids back into constitutive expression plasmids so that they can also be used in non-Cre-expressing cells and in non-genetically modified animal models. Therefore developing a universal protocol for this purpose is useful as it could save a lot of valuable time and lab resources.

**Result:**

Here we report a simple, quick, and cost-efficient protocol to invert the orientation of the open reading frame (ORF) within FLEX switch containing plasmids using commercial Cre recombinase. This protocol, requiring as little as 30 min and 50 ng of plasmid, has a cloning efficiency of 40–50%. To our surprise, single step recombination efficiency between the two mutant Lox2272 sites turned out very low. To understand this, we performed in vitro recombination assays. These assays revealed, significant impairment in recombination between Lox2272 sites as compared wild type LoxP sites in the FLEX plasmids.

**Conclusion:**

We have presented an in vitro protocol to invert the ORF in FLEX based plasmids. This protocol is simple and highly efficient. Thus this method expends current molecular biology toolbox. We also demonstrate that the recombination between Lox2272 sites is much less efficient than wild type LoxP sites. This result has important implication for evaluating the efficacy of FLEX switch in biological systems and provides a rationale for future development of higher efficiency LoxP mutants.

## Background

The Cre-LoxP system, derived from P1 bacteriophage, is a site-specific recombination system extensively used in experimental organisms [1, 2]. In this genetic binary system, Cre recombinase catalyzes site-specific recombination between two LoxP sites, which are 34 bp DNA fragment containing an asymmetric 8 bp (base pair) spacer flanked by two 13 bp inverted repeats [1].

Outcomes of the Cre-mediated recombination are determined by the orientations of two LoxP sites. When the two LoxP sites are arranged in same orientation, the Cre recombinase can excise any intervening sequence flanked by LoxP sites. On the other hand when the two LoxP sites are placed in the reciprocal orientation, Cre recombinase can inverse the sequence between the two LoxP sites. Finally, when the two LoxP sites are placed on two different DNA molecules, recombination of intermolecular LoxP sites can lead to site-specific insertion or reciprocal translocation between the two DNA [3, 4].

In 2003, a novel application of Cre/LoxP system called the FLEX switch was invented [5]. Briefly, the FLEX switch involves two wild type LoxP sites and two mutant LoxP sites such as Lox511 or Lox2272. The elegant design allows two pairs of mismatched LoxP sites to first invert and turn on the switch, and a subsequent excision event to eliminate one of the LoxP partners to prevent re-inversion. The method greatly reduces leaky expression in the absence of Cre recombinase, making the FLEX switch the preferred method to turn on genes in mammalian cells. In particular, FLEX switches have been extensively used in recombinant Adeno-associated virus (rAAVs) vectors to spatially restrict gene delivery to cells expressing Cre recombinase [6–10]. To date, countless of FLEX switch plasmids have been generated. Several hundreds of them can be found in addgene alone *(*https://www.addgene.org/).

Recently we found crucial needs in our experiments to reverse ORF in several FLEX plasmids so that we can express gene of interest constitutively in a non-genetically modified animal model. We initially obtained these FLEX plasmids from Addgene and our goal was was to flip ORF only, while maintaining other features of the parental FLEX plasmids, such as the promoter and viral vector components. However we realized it might take many days or even weeks to produce such constructs by using conventional cloning methods. Moreover lack of suitable restriction sites for cloning from our DNA templates would make the cloning task more difficult. To overcome these hurdles we set out to test whether we can simply generate the reverted form in vitro by taking advantage of the quadruple LoxP sites within the FLEX plasmids. We explored the experimental conditions for flipping back ORF in FLEX plasmid and we tested cloning efficiency systematically. Here in this report we demonstrate simple and practical protocols for achieving in vitro Cre dependent inversion of ORF within FLEX based plasmid (workflow is illustrated in Fig. 8).

## Results

### Cre recombinase effectively inverted ORF in FLEX plasmid in vitro

Although conventional molecular cloning approach can be taken to reverse the ORF in FLEX-based plasmids, this approach can be time consuming and labor intensive. Therefore we decided to test whether a more straightforward and efficient in vitro protocol could flip the ORF with commercially available Cre recombinase (*New England Biolabs*).

**Fig. 1:**
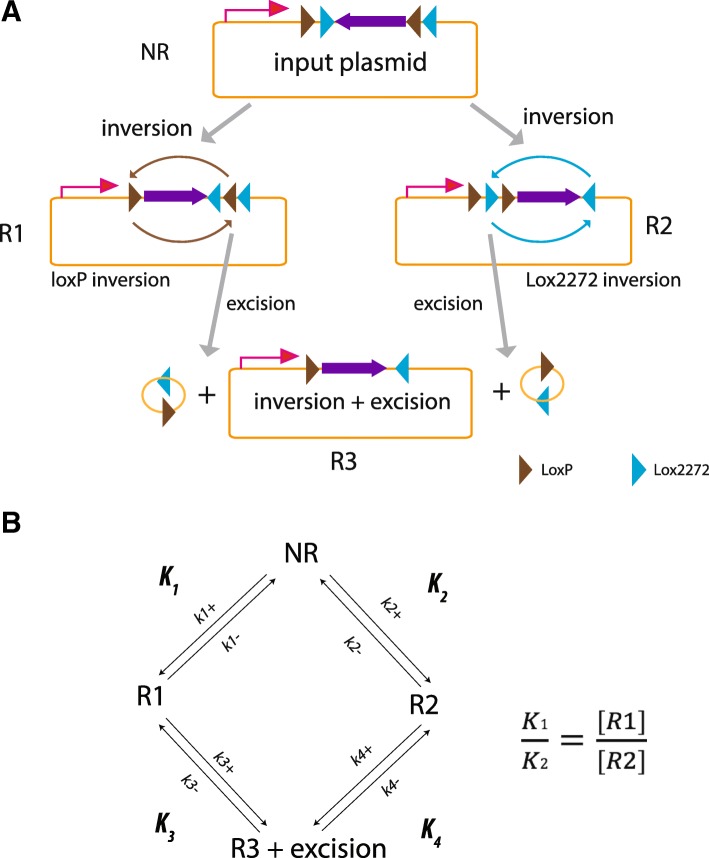
Scenarios of in vitro flip of ORF in FLEX plasmid. **a** Cre-mediated recombination. Starting from input plasmid, Cre enzyme can induce ORF inversion via two LoxP sites (R1) or two Lox2722 sites (R2). Excising linker sequence between two Lox sites of the same orientation in either R1 or R2 yields R3. Purple triangles, LoxP sites; Blue triangles, Lox2272 sites. **b** Diagram of Cre-mediated reactions at equilibrium

As illustrated in Fig. 1a, our input templates carry a pair of wild type (WT) LoxP sites and two Lox2272 sites [11] both in a head to head configuration. Recombination catalyzed by Cre could have four outcomes: 1) no recombination (NR), 2) WT LoxP site inversion (R1), 3) Lox2272 inversion (R2), or 4) either R1 or R2 followed by excision (R3). Because recombination is reversible, these four reactions tend to approach equilibrium between substrates and reaction products. Consequently full recombination will never be achieved (Fig. 1b). Therefore our initial concern was that the efficiency of recombination under in vitro conditions might not be high enough to obtain desired products with minimal screening efforts. However all three recombination products (R1–3) could be undertaken as they all have their ORF reversed (Fig. 1a).

**Fig. 2:**
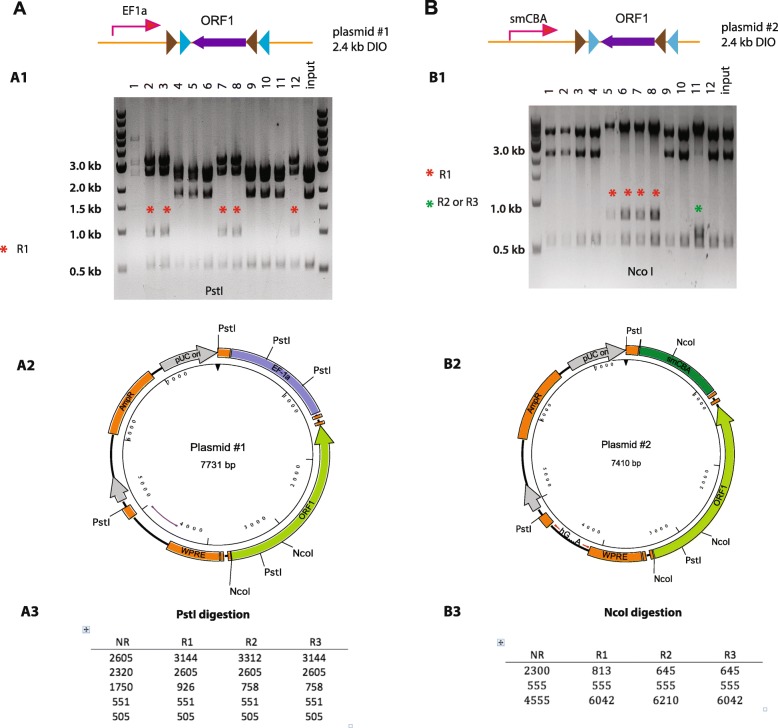
Cre recombinase effectively inverted ORF in FLEX plasmid in vitro. **a** Inversion of ORF in plasmid #1. Top, plasmid diagram. (A1) PstI screening. A 926 bp band by pst1 digestion of R1 can be seen form 5 plasmids (# 2,3,7,8,12). There were no R2 and R3 plasmids (should yield a 758 bp band). Colony #1 had low yield. (A2) Plasmid Map. Shown are five PstI sites and two NcoI sites. (A3) Prediction of PstI digestion. **b** Inversion of ORF in plasmid #2. Top, plasmid diagram. (B1) NcoI digestion. An 813 bp band by NcoI digestion of R1 can be seen from 4 plasmids (# 5,6,7,8). #11 could be either R2 or R3 plasmids for the presence of 645 bp band. (B2) Plasmid Map. PstI and NcoI sites are shown. (B3) Prediction of NcoI digestion

To determine the efficiency of the in vitro inversion reaction we performed three separate experiments by using different FLEX plasmids and different conditions. Table 1 listed all plasmids we used in this study. In the first reaction, we used a plasmid with an ORF construct flanked by quadruple LoxP sites (plasmid #1, 2.4 kb DIO) (Fig. 2a). We incubated 250 ng of plasmid DNA with 1 μl (1 unit/μl) of Cre recombinase for 30 min at 37 **°**C. The Cre recombinase was then heat inactivated (10 min at 70 °C) and the whole reaction volume (15 μl) was used to transform Stbl3 competent cells. From the agar plate we picked 12 colonies which were further expanded before isolation of the plasmid using a standard mini-prep protocol. To determine whether plasmids from each of the 12 samples had undergone Cre mediated inversion of the ORF we performed restriction enzyme digest using enzymes that enable us to distinguish the different potential recombination products. For example, there are 5 PstI sites in the plasmid #1. Two of them are located in the Ef1a promoter. One is in the ORF. The remaining two PstI sites are in the plasmid backbone (Fig. 2 A2). Using PstI digestion, we can distinguish each of recombination products (Fig. 2 A3). Testing plasmids samples by digestion with PstI, we found that 5 out of 11 samples had the pattern signifying the flipped ORF (Fig. 2a). The plasmid #1 had low yield although it appeared to be R1. To our surprise, there were no R2 and R3 plasmids in this screen, suggesting that inversion at two Lox2272 sites was not efficient. Nevertheless, this in vitro reaction achieved a very prominent efficiency (5 out of 12).

**Table 1:** List of plasmids used in this study

list of plasmids						
Plasmid	Name	Size of DIO or SIO (kb)	Gene of interest	Promoter	Enzymes for cloning experiments	Enzyme for in vitro experiments
#1	pAAV-Ef1a-DIO-ORF1	2.4	ORF1	smCBA	PstI,	XhoI, BsrGI, NsiI
#2	pAAV-smCBA-DIO-ORF1	2.4	ORF1	Ef1a	NcoI, AgeI, XhoI	
#3	pAAV-EF1a-DIO-EGFP	0.9	EGFP	Ef1a	NheI, EcoRI, BamHI,	XhoI, BsrGI
#4	pAAV-Ef1a-DIO-tdtomato	1.5	tdTomato	Ef1a		BamHI, XhoI, BstXI
#5	pAAV-Ef1a-DIO-ORF2	1.7	ORF2	Ef1a		BamHI, EcoRV
#6	pAAV-Ef1a-SIO(loxP)-ORF1	2.4	ORF1	Ef1a	PstI	PstI
#7	pAAV-Ef1a-SIO(lox2272)-ORF1	2.4	ORF1	Ef1a	PstI	PstI

In a separate experiment we used an alternate FLEX plasmid with same ORF insert (DIO 2.4 kb) but different promoter (plasmid #2) (Fig. 2b). For this reaction we reduced the amount of input plasmid (50 ng) and extended the reaction time to 1 h at 37 **°**C. The promoter in plasmid #2 does not contain PstI site, making PstI screening less useful (Fig. 2 B2). However, the presence of three NcoI sites, with one located in the promoter region and two located in the 5′ region of the ORF, allowed for an effective NcoI screening (Fig. 2, B2–3). We again screened 12 colonies. Testing recombination products by digestion with NcoI, we found that four colonies were R1 (#5,6,7,8). Colony #11 was further determined as R3 by Age1/Xho1 enzyme combination (data not shown). Therefore, the overall recombination efficiency (5 out of 12) in this experiment was similar to the last one. Once again, R1 remained the predominant recombination products and there was no R2 plasmid.

**Fig. 3:**
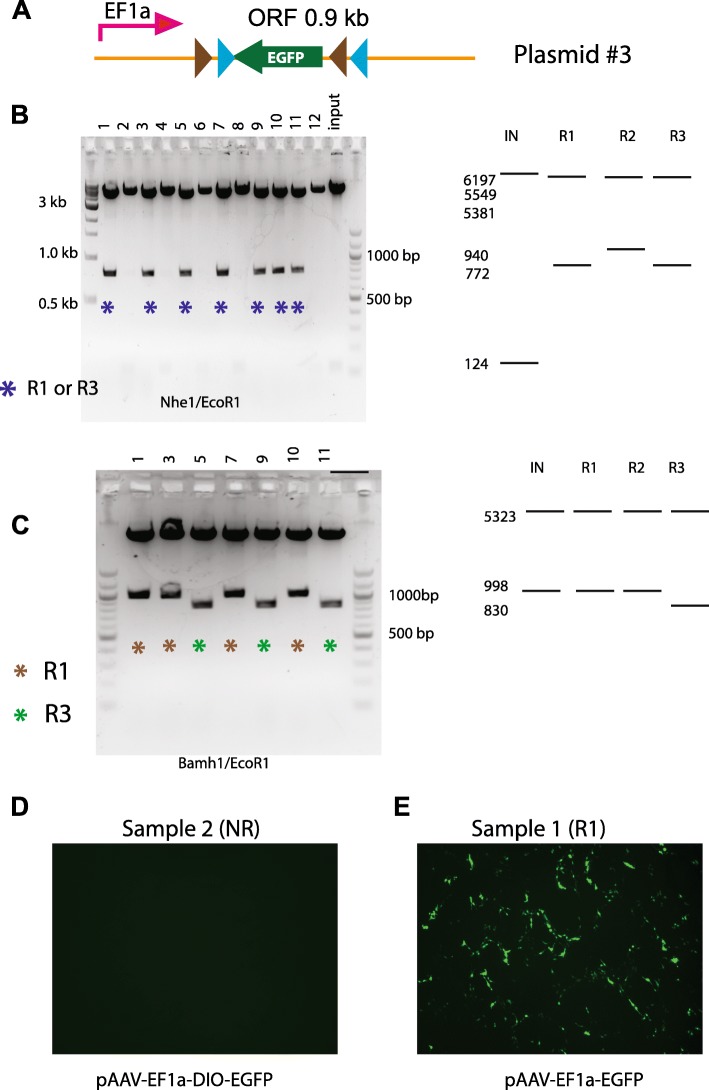
Higher numbers of two-step recombination was obtained from a plasmid with shorter DIO sequence. **a** Diagram of input plasmid. **b** NheI/EcoRI digestion. Seven plasmids (#1,3,5,7,9,10,11) have flipped ORF as they showed band of 772 bp. These plasmids could be either R1 or R3 according to predicted patterns (right panel). **c** BamhI/EcoRI digestion. Bands of 998 bp and 830 bp were yielded from R1 (#1,3,7,10) and R3 (#5,9,11), respectively. Right panel, predicted patterns. **d-e** Example images of HEK cells transfected with plasmid # 2 (NR) (**d**) and #1(R1) (**e**). As expected, fluorescence was detected in **e**, but not in **d**

In a third experiment we attempted to flip an EGFP ORF in a pAAV-EF1a-DIO-EGFP plasmid (plasmid #3) (Fig. 3). The DIO sequence in the plasmid #3 is shorter (0.9 kb) than #1 and #2. In addition, we can confirm the functionality of the plasmids once the ORF is flipped by GFP fluorescence. The reaction was performed using an even shorter reaction time (20 min) with 50 ng of plasmid and 1 unit of Cre. Testing plasmids by digestion with NheI/EcoRI (Fig. 3b), we found that 7 out of 12 had the flipped ORF and their digestion patterns were consistent with them being either R1 or R3. Further test with Bamh1/EcoR1 digestion established four out of the 7 (#1,3,7,10) as R1 while the other three (#5,9,11) as R3 (Fig. 3c). Transfecting HEK293 cells with DNA from each of the samples allowed us to confirm the results of the restriction digests, with GFP labeled cells were observed from transfections with forward orientation EGFP plasmids (Fig. 3e), and no fluorescence from the reverse orientation (Fig. 3d). Therefore this experiment using shorter DIO sequence yielded a marginally higher percentage of recombination events (7 out of 12). Despite that we obtained higher number of double recombinant R3 products (3 out of 12), there was still no R2 found in this screen.

### Effects of DIO sizes on cloning efficiency

**Fig. 4:**
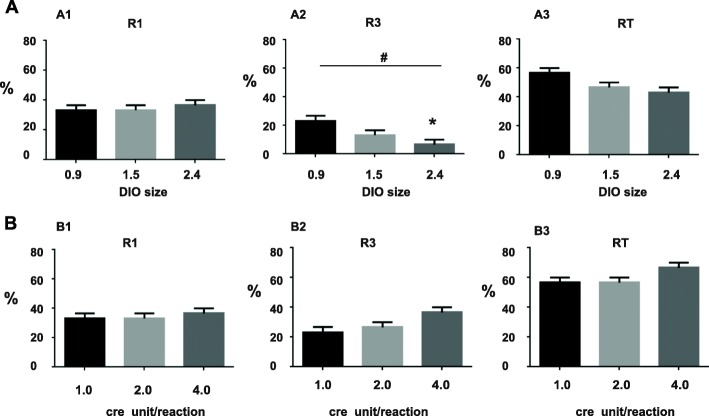
Effects of DIO sizes and concentrations of Cre on cloning efficiencies. **a** Effects of DIO sizes. Bar charts showing the percentage of R1 colonies (A1), R3 colonies (A2) and total recombinants (RT) (A3). The results are averaged from three independent experiments. Bars indicate the S.E.M. #, *p* < 0.05, one way ANOVA; *, *p* < 0.05, Tukey multi-comparison test. **b** Effects of Cre concentrations. Bar charts showing the percentage of R1 colonies (B1), R3 colonies (B2) and RT (B3). The results are averaged from three independent experiments. Bars indicate the S.E.M

In order to examine more carefully whether the length of DIO in plasmids affects cloning efficiency, we compared three plasmids (#3, #4, #1) of different DIO sizes (0.9 kb, 1.5 kb, 2.4 kb respectively) using an identical condition (250 ng DNA treated with 1unit of Cre enzyme in a total volume of 15 μl for 30 min). This condition is hereinafter referred to as “standard condition”. After cre reaction and transformation, 10 transformants were randomly chosen for screening. Restriction digest and DNA electrophoresis were performed to determine the identity of each transformant. Screenings of #1 and # 3 have been described previously. Screening of # 4 was conducted using Bamh1, XhoI and BstXI. Three independent experiments were run for each plasmid template. Results were summarized in the bar graphs in Fig. 4a. Again, no R2 was found. There was no difference in the efficiency of obtaining R1 (One way ANOVA, F_2,6_ = 0.33,*p* = 0.73)(Fig. 4 A1). However, cloning efficiency of R3 was different among templates, with the plasmid of shorter DIO yielding significantly more R3 (Fig. 4 A2) (One way ANOVA, F_2,6_ = 6.33, *p* = 0.03; Tukey’s multiple comparisons test, *p* < 0.05 for DIO 2.4 vs DIO 0.9). Overall cloning efficiency (RT), which include both R1 and R3, was not different among the three groups (One way ANOVA, F_2,6_ = 4.33, *p* = 0.07).

### Effects of increasing concentrations of Cre enzyme on cloning efficiency

Next, we tested whether we can further boost cloning efficiency by increasing the concentrations of cre recombinase. Here plasmid #3 was taken as an example. Three different concentrations of enzyme (1, 2 and 4 units) were compared using the standard condition (250 ng DNA, 30 min). All recombination products were found to be either R1 or R3. Statistic analysis showed that Increasing Cre enzyme had no effect on R1 (Fig. 4 B1) (One Way ANOVA, F_2,6_ = 0.33, *p* > 0.05) and RT (Fig. 4 B3) (One Way ANOVA, F_2,6_ = 3.0 *p* > 0.05). Although increasing Cre appeared to lead to more R3 (Fig. 4 B2), the group difference did not reach significance (One Way ANOVA test, F_2,6_ = 4.33, *p* = 0.068).

### Recombination between Lox2272 sites is less efficient than wild type LoxP sites

The foregoing experiments demonstrated that in vitro application of Cre could efficiently flip ORF of FLEX plasmid. However it is surprising that in all cloning experiments we did not obtain a single R2 colony. This result strongly implied that recombination efficiency between Lox2272 is lower than that of wild type LoxP sites. To test this hypothesis, we performed in vitro assays using different FLEX plasmids. Because LoxP and Lox2272 sites are located in the same plasmid, it offers a unique opportunity for us to directly compare their recombination efficiencies. Our aim through these experiments is to estimate fractions of each recombination products and to compare efficiencyof recombination between the two wild type LoxP sites and the two Lox2272 sites.

**Fig. 5:**
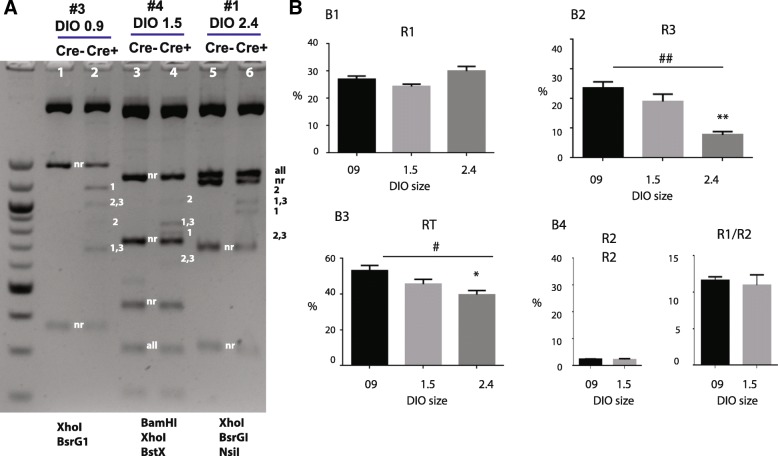
In vitro recombination assays: Effects of DIO sizes. **a** Example gel image of in vitro recombination assay. Three plasmids of different DIO sizes were examined. Each was digested with different restriction enzyme combinations in order to distinguish different recombination products. Plasmid # 3 (DIO 0.9) was digested with XhoI and BsrG1. Plasmid # 4 (DIO 1.5) was digested with BamHI, XhoI and BstXI. Plasmid # 1 (DIO 2.4) was digested with XhoI, BsrGI and NsiI. DNA bands for different products are marked. 1, 2, 3, nr represent R1, R2, R3, non-Recombination respectively. **b** Bar charts showing the percentage of R1 (B1), R3 (B2) and RT (B3). (B4) Comparisons of R2 (left) and R1/R2 ratios (right) between plasmids #3 (0.9 kb DIO) and plasmids # 4 (1.5 kb DIO). The results are averaged from three independent experiments. Bars indicate the S.E.M. #, *p* < 0.05, ##, *p* < 0.01, one way ANOVA; *, *p* < 0.05, **, *p* < 0.01, Tukey multi-comparison test

In the first set of experiments (Fig. 5), we compared three plasmid carrying different lengths of DIO (# 3, #4 and #1). Cre reactions were conducted using the standard condition. At completion, DNA samples cut with appropriate endonucleases combinations, followed by electrophoresis, to determine the fraction of each recombination products. Endonucleases combinations were chosen with an aim to distinguish many recombination products in single reaction. Therefore they differ from those used in the cloning experiments (Table 1).

For the plasmid with 0.9 kb DIO (plasmid #3), we used BsrGI and XhoI endonucleases (Fig. 5, lanes 1,2). This enzyme combination is predicted to yield 375 bp and 1437 bp bands for NR, 683 bp and 1165 bp for R1, 851 and 997 bp for R2, and lastly, 683 bp and 997 bp for R3. As expected, the control experiment without Cre produced only NR bands (Lane 1). In contrast, several recombination bands can be detected in the sample treated with Cre (Lane 2). Not surprisingly, R2 specific band (851 bp) was much fainter than the R1 band (1165 bp), the R1-R3 band (683 bp) and the R2-R3 band (997 bp). We used Bio-Rad’s Image Lab software to get semi-quantitative analysis of different fractions in our sample. To estimate DNA molar quantities more accurately, intensities of DNA bands were divided by the sizes of DNA to convert intensities to DNA molar amounts. Using this analysis, we calculated that R1 and R3 each accounted for ~ 28% and ~ 23% of total DNA. About 45% DNA did not undergo recombination.

Plasmid containing 1.5 kb DIO (plasmid #4) (Fig. 5, lane 3, 4) was digested by BamHI, Xho1 and BstX1. This combination of enzymes can produce distinct patterns for NR (1303, 726, 448, 316 and 220 bp), R1 (836, 783, 726, 448 and 220 bp), R2 (1004, 726, 615, 448 and 220 bp) and R3 (836, 726, 615, 448 and 220 bp). Compared with the Cre- sample (lane 3), the Cre + sample (lane 4) showed several extra bands including a 783 bp R1 band, a 836 bp R1-R3 band, a weak 615 bp R2-R3 band and an even weaker 1004 bp R2 band. Quantitative gel analysis revealed that R1 and R3 accounted for 24% and 19% of total DNA respectively.

For the plasmid with 2.4 kb DIO (plasmid #1), we cut DNA with Xho1 BsrG1 and Nsi1. This combination should produce a 1329 bp band for all products, a 1236 bp band for NR, a 1169 bp band for R2, a 1001 band for R1 and R3, a 928 bp band for R1, a 760 bp band for R2 and R3, a 701 bp band or NR and a 332 bp band for NR. In the example (Fig. 5, lane 6), the 1001 bp R1-R3 band and 928 bp R1 band and were visible. The 760 bp R2-R3 band was very weak. The predicted 1169 bp R2 band was too faint for gel detection.

Averaged fractions of R1, R3, and RT for each DNA template are shown in the bar charts in Fig. 5b. Statistic analysis found no difference in R1 among the three templates (One way ANOVA, F_26_ = 17.08, *p* = 0.003) (Fig. 5 B1). However, R3 was significantly lower in plasmids with large DIO (F_26_ = 17.08, *p* < 0.01) (Fig. 5 B2). Total fraction of recombination (RT), which was calculated by 1 - NR%, was also significantly affected by DIO sizes (F_26_ = 6.14, *p* < 0.05) (Fig. 5 B3). Thus, shorter DIO favors R3 and RT.

We tried to estimate R2 fractions from all DNA templates. However R2 bands in plasmid #1 (DIO 2.4 kb) were too weak for measurement. Therefore analysis of R2 in plasmid #1 was omitted. For plasmid #3 and #4, their R2 fractions were estimated to be less than 2.5% of total DNA (Fig. 5 B4, left). We calculated ratio of R1/R2 for plasmid #3 (11.6 ± 0.85) and # 4 (11.0 ± 2.4) (Fig. 5 B4, right). Based on these numbers, we decide that equilibrium constant (K) of recombination between two LoxP sites is about 10 times higher than that of Lox2272 sites. However, cautions must be taken in evaluating K1/K2, mostly because measuring R2 is quite subject to error due to its low intensity. In spite of this concern, the disparity between the R1 band and R2 band is obvious.

### Time course of Cre-mediate reaction

**Fig. 6:**
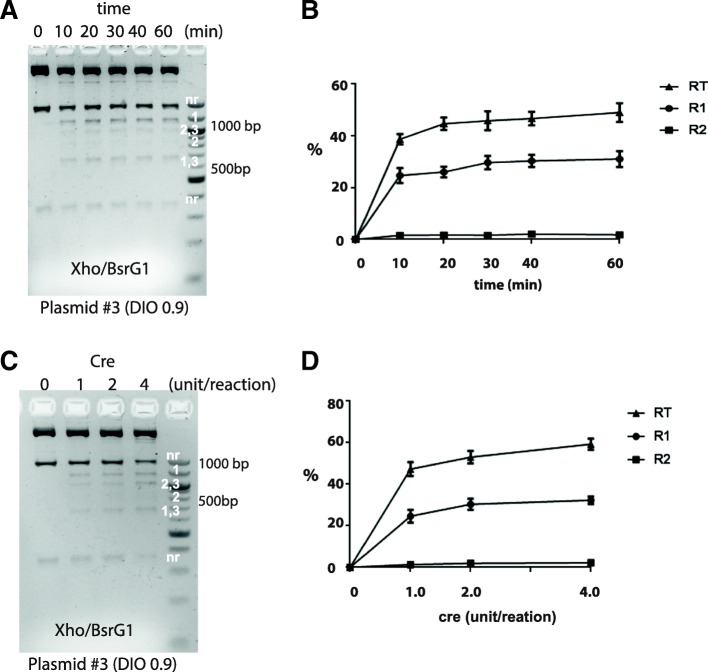
In vitro recombination assays: Time course and Cre enzyme dose effect. **a-b** Time course. **a** Example gel image. DNA was digested by XhoI BsrGI. DNA bands for different products are marked. **b** Time course plot illustrating the change in recombination products over the time. The results are averaged from three independent experiments. Error bars mean S.E.M. **c-d** Effects of increasing Cre on recombination. **c** Example gel image. **d** Plot of recombination products as function on cre concentrations. The results are averaged from three independent experiments. Error bars mean S.E.M.

Next we decided to establish the time course of Cre-mediate reaction more precisely and to determine whether compositions of recombination products differ at different time point. Plasmid #3 (0.9 kb DIO) was chosen as an example. Briefly 250 ng DNA was incubated with Cre (I unit) for a given time and then reaction was stopped by heat inaction, followed by BsrGI/XhoI digestion. Experiments were performed in triplicate. A gel example is shown in Fig. 6a. At beginning, there were only NR bands but no recombinant products. However 10 min after reaction, the R1 and R1-R3 bands already became prominent whereas the R2 band (851 bp) was barely detectable. After reaction reached 20 min, additional time did not appreciably increased the recombination products, suggesting that reaction was close to equilibrium at this time point. Meanwhile patterns of DNA digestions were also largely unchanged after 20 min through which time the R2 bands remained very weak (Fig. 6a). Results of quantitative gel analysis are summarized in a time-course plot in Fig. 6b. As clearly illustrated, the in vitro reaction took very short time to approach equilibrium. About 40–50% of DNA plasmids have undergone rearrangements. Meanwhile R2 fraction was low (less than 2.5%) through out the experiments.

### Effects of increasing Cre enzyme on recombination efficiency

Plasmid #3 was again used as an example for this test (Fig. 6c, d). Reactions were carried out in the standard condition. As the cloning experiment (Fig. 3b), we examined 3 conditions where Cre enzymes for each reaction were 1, 2 and 4 units respectively. Overall, digestion patterns were similar at different Cre concentrations. R1, R1-R3 and R2-R3 bands were visible whereas R2 bands (851 bp) are weak even at higher Cre lanes (Fig. 6c). Increasing Cre from 1 unit/reaction did not produce a large effect, as statistical analysis found no significant difference in fractions of R1 (One way ANOVA, F_2,6_ = 2.25, *p* = 0.18), R2 (One way ANOVA, F_2,6_ = 2.48, *p* = 0.16) and RT (One way ANOVA, F_2,6_ = 3.92, *p* = 0.08) at three Cre concentrations (Fig. 6d).

### Cre-mediated inversion of DIO was not sequence dependent

**Fig. 7:**
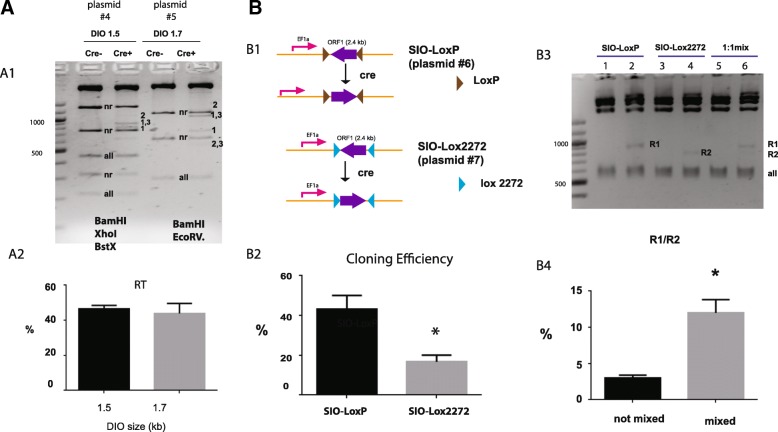
In vitro recombination assays: Effects of DIO-flanked sequence (**a**) and inversion of ORF in SIO plasmids (**b**). **a** Inversion of ORF in DIO is not sequence dependent. Two plasmids (#4 and #5) with similar DIO sizes were compared. (A1) Example gel. Plasmid #4 was digested with BamHI, XhoI and BstXI. Plasmid #5 was digested with BamHI and EcoRV. (A2) Comparison of total recombination products (RT) between the two DIO plasmids. The results are averaged from three independent experiments. Error bars mean S.E.M. **b** Cre mediated inversion of ORF in SIO plasmids. (B1) Diagrams of SIO plasmids and Cre-mediated inversion. (B2) Comparison of the rate of ORF inversion between the two SIO plasmids. Bar chart shows the percentage of colonies with ORF inversion. Results are averaged from three independent screens. (B3) Example gel image. In vitro reactions were conducted using the standard protocol. SIO plasmids were cut with PstI. Lane 1, 3, 5 were control samples without Cre treatment. Lane 2, 4, 6 were samples with Cre treatment. The 824 bp band 740 bp band representing recombination products of two SIO plasmids are marked by R1 and R2 respectively. (B4) Bar chart of R1/R2 ratios. The results are averaged from three independent experiments. Error bars mean S.E.M. *, t-test, *p* < 0.05

To test whether Cre-mediated recombination in FLEX plasmid is sequence dependent, we performed in vitro recombination assays (Fig. 7a) using two DIO plasmids (# 4, 1.5 kb DIO and # 5, 1,7 kb DIO) that have similar DIO sizes but completely different DIO-flanked sequences (Table 1). Plasmid #4 was digested with BamH1, XhoI and BstX1. Plasmid # 5 was digested with EcoRV and BamH1. For both templates, R1 bands and R1-R3 bands were quite prominent in Cre + lanes. In contrast, R2 bands were barely detectable in these lanes (Fig. 7 A1). Quantitative analysis of NR bands allowed us to derive the fractions of total recombination products (RT). For both templates, about 50% of total DNA underwent recombination (Fig. 7 A2). There was no difference between the two DNA templates (t-test, *p* > 0.05), suggesting that in vitro Cre recombination is not sequence dependent.

### Cre mediated inversion in the single inverted open reading frames (SIO) plasmids

We wondered whether presence of the two loxP sites in the FLEX plasmid could interfere recombination between the two Lox2272 sites. To test this, we constructed two new plasmids (# 6 and # 7) from plasmid #1. They each contain two Lox sites arranged in the inverted orientation (Single-floxed inverted ORF, SIO) (Fig. 7 B1). Upon recombination, flanked sequence in these plasmids will be inverted (Fig. 7 B1).

To test the efficiency of inversion, we treated these plasmids with Cre (250 ng DNA, 1 unit of Cre for 30 min) and then transformed them into bacteria. We screened colonies using PstI digestion. This digestion is predicted to produce a distinct 824 bp band from an inverted SIO-loxP plasmid and a 740 bp band from an inverted SIO-lox2272 plasmid. We found that inversion frequency was much higher in SIO-loxP plasmids (43%) than that in SIO-lox2272 plasmids (16%) (Fig. 7 B2) (t-test, *p* < 0.05). To confirm this cloning result, we conducted in vitro assays. We used PstI digestion again to resolve the recombination and non-recombination products. As expected, an 824 bp band (R1) was generated from SIO-loxP after Cre treatment (Fig. 7 B3, lane 2). A 740 bp band (R2) from SIO-Lox2272 was also visible, although it was weaker (Fig. 7 B3, lane 4). Ratio of normalized R1 vs R2 was 2.97 ± 0.43 (Fig. 7 B4, non-mixed sample). This number was consistent with the result of plasmid screening (Fig. 7 B2). Interestingly, when we mixed equal amount of two plasmids (125 ng of each) and treated the mixture with cre, we observed a much weaker R2 band (Fig. 7 B3, lane 6) and much larger R1 vs R2 ratio (Fig. 7 B4, paired t-test, *p* = 0.02). Therefore in the presence of LoxP sites, recombination between Lox2272 sites became even less efficient.

## Discussion

**Fig. 8:**
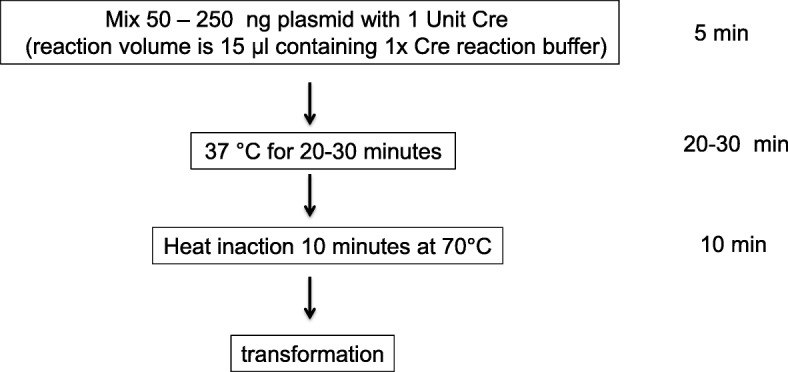
Protocol overview. The input plasmid is first mixed with Cre enzyme in 1× reaction buffer. After 20–30 min of incubation at 37 **°**C, the reaction is brought to 70 **°**C for 10 min to heat inactivate Cre recombinase. The whole reaction can then be directly used for transformation

Since the original publication of the FLEX design [5], a significant increasing number of FLEX plasmids have been generated. Therefore developing a simple method to convert these plasmids back to plasmid that can express gene of interest constitutively is quite worthwhile. An important advantage of using Cre mediated in vitro inversion is that it is without requirement of specific restriction recognition sites and thus can be universally applied to any FLEX-based plasmid. The second advantage of the in vitro Cre recombination method is that it is simple and cost-effective. A simple workflow of protocol is illustrated in Fig. 8. This protocol takes only a few minutes to set up, can have plasmid ready for transformation within 45 min and carries minimal cost.

It must be noted that the idea to invert the Double-floxed Inverted ORF in a plasmid was previously demonstrated to work in vitro in the original study [5]*.* However it has not been widely publicized as we found no further report since then to use this method to generate new plasmid constructs. The in vitro experiment in the initial study was designed primarily to demonstrate the Cre-mediated rearrangements instead of cloning efficiency [5] and therefore the authors used fairly large amount of crude Cre extract (100 μl) and plasmid (3 μg) in their protocol which may have discouraged its application [5].

Mutant LoxP sites have been developed in order to expand the functional applications of Cre/LoxP systems. They fall into two classes: spacer mutants and inverted-repeat mutants [12, 13]. The spacer mutants such as Lox5171, Lox2272 [11] and Lox511 [14] carry mutation within the 8 bp spacer region of the LoxP sites. These mutations prevent them from recombine to wild type LoxP sites but they can readily recombine with themselves. This special property makes them suitable for the FLEX constructs. However, the recombination efficiencies of LoxP spacer mutants were rarely discussed as many researchers assume that these mutant LoxP sites have similar recombination efficiency as wild type LoxP sites. To our surprise, we found that recombination between Lox2272 sites was much less efficient than wild type LoxP sites. Interestingly, the difference was smaller in the SIO plasmids (Fig. 7b) than in the DIO plasmids, suggesting that presence of wild type LoxP sites inhibits recombination between the mutant sites. This notion is consistent with the classic model of enzyme inhibition in which LoxP sites could serve as competitive inhibitors for Cre reactions using Lox2272 sites as substrate. Thus quadruple arrangement of the LoxP sites and Lox2272 sites in FLEX plasmids allows a new way to assess recombination efficiency and possible interference between LoxP and Lox2272 sites. This strategy may be extended to examine other spacer mutants such as Lox5171 and Lox522. Ultimately, our result has implications in evaluating effectiveness FLEX switch as lower recombination efficiency in Lox2272 in the FLEX plasmids is anticipated to compromise the effects of Cre-mediated gene activation. Thus future development of additional LoxP spacer mutants with higher recombination efficiency should be beneficial.

## Conclusion

We have presented an in vitro protocol to invert the ORF in FLEX/DIO based plasmids. This protocol is simple, taking only a few minutes to set up, quick, (reaction times can be as short as 20 min), low cost (1 μl of enzyme per reaction, $1–2) and highly efficient (up to 50% positive colonies). Thus this method expends molecular biology toolbox. We show our workflow in Fig. 8. In addition, we provide direct evidence that recombination between Lox2272 is about 10 times less efficient than wild type LoxP sites in the FLEX plasmids.

## Methods

### Bacterial strains, plasmids and culture conditions

All the plasmids used in this study carry ampicillin resistant gene β-Lactamases. The plasmids were transformed and maintained in *Escherichia coli* strains Stbl3 (Invitrogen™). *Escherichia coli* were grown at 37 **°**C in Luria Bertani (LB) medium supplemented with ampicillin at 100 μg/ml.

### Molecular biology methods

Plasmid DNA mini-preparations were performed using QIAGEN kits (QIAGEN). Standard DNA electrophoresis was performed to capture patterns of restriction digest of different plasmids. Restriction endonuclease and Cre recombinase at cost of $68 for 50 units in one vial were purchased from New England Biolab (Beverly, MA). Quantification of DNA intensities was carried out with Bio-Rad’s Image Lab.

### In vitro Cre recombination reaction

Cre recombination reactions were conducted in 1× Cre buffer containing 33 mM NaCl, 50 mM Tris-HCl,10 mM MgCl2 (pH 7.5 @ 25 °C). Experiments carried out at different conditions have been compared. . Generally, 50–250 ng of plasmid DNA were treated with 1–4 units (1 unit/μl) of Cre recombinase enzyme in 15 μl reaction volume at 37 °C for 20–60 min. The Cre recombinase was then heat inactivated (10 min at 70 °C) and the whole reaction volume was used to transform Stbl3 competent cells or for *the* in vitro Cre recombination assays.

For the for in vitro Cre recombination assay, DNA was cut with appropriate restriction enzymes. The digested DNA was then subjected to gel electrophoresis and DNA bands were detected by ethidium bromide staining.

### Equations

Scheme of Cre-mediated reaction is shown in Fig. 1b. The equations below contend with the forward and reward reaction containing NR (No Recombination). Because at equilibrium, forward and reverse reactions occur at equal rates, we have:

1 $$ {\left[ NR\right]}^{\ast }{k}_{1+}={\left[R1\right]}^{\ast }{k}_{1-} $$

2 $$ {\left[ NR\right]}^{\ast }{k}_{2+}={\left[R2\right]}^{\ast }{k}_{1-} $$

The equilibrium constant (*K*), which is defined as ratio of forward reaction constant vs reverse reaction constant (*k*_+_/*k*_−_), can thus be derived as the ratio of recombination products vs. NR:

3 $$ {K}_1=\frac{k1+}{k1-}=\frac{\left[R1\right]}{\left[ NR\right]} $$

4 $$ {K}_2=\frac{k2+}{k2-}=\frac{\left[R2\right]}{\left[ NR\right]} $$

Because [NR] is the same for the two reactions, we can then derive our final equation:

5 $$ \kern0.5em \frac{K_1}{K_2}=\frac{\left[R1\right]}{\left[R2\right]} $$

### Data analysis

Statistical analyses were conducted with Graphpad Prism software. Two sample comparisons were made using an Student’s *t*-test. For multiple comparisons, One-way ANOVA was used followed by post-hoc Tukey’s multi-comparison test. Differences were considered significant when *p* < 0.05. Data are shown as mean ± SEM.

## Abbreviations

bp: Base pair
DIO: Double-floxed Inverted Open reading frame
ORF: Open reading frame
